# Modulation of Rolandic Beta-Band Oscillations during Motor Simulation of Joint Actions

**DOI:** 10.1371/journal.pone.0131655

**Published:** 2015-07-07

**Authors:** Mathilde Ménoret, Mathieu Bourguignon, Riitta Hari

**Affiliations:** 1 Brain Research Unit, Department of Neuroscience and Biomedical Engineering, Aalto University, Aalto, Finland; 2 Laboratoire sur le Langage, le Cerveau et la Cognition (L2C2), UMR5304, CNRS/UCBL, Lyon, France; 3 MEG Core, Aalto NeuroImaging, Aalto University, Aalto, Finland; Università di Trento, ITALY

## Abstract

Successful joint actions require precise temporal and spatial coordination between individuals who aim to achieve a common goal. A growing number of behavioral data suggest that to efficiently couple and coordinate a joint task, the actors have to represent both own and the partner’s actions. However it is unclear how the motor system is specifically recruited for joint actions. To find out how the goal and the presence of the partner’s hand can impact the motor activity during joint action, we assessed the functional state of 16 participants’ motor cortex during observation and associated motor imagery of joint actions, individual actions, and non-goal-directed actions performed with either 1 or 2 hands. As an indicator of the functional state of the motor cortex, we used the reactivity of the rolandic magnetoencephalographic (MEG) beta rhythm following median-nerve stimulation. Motor imagery combined with action observation was associated with activation of the observer’s motor cortex, mainly in the hemisphere contralateral to the viewed (and at the same time imagined) hand actions. The motor-cortex involvement was enhanced when the goal of the actions was visible but also, in the ipsilateral hemisphere, when the partner’s hand was visible in the display. During joint action, the partner’s action, in addition to the participant’s own action, thus seems to be represented in the motor cortex so that it can be triggered by the mere presence of an acting hand in the peripersonal space.

## Introduction

Joint actions are ubiquitous in our normal social life: we shake hands, carry heavy objects and play games with others. Such actions are typically easy and natural to perform although they require precise temporal and spatial coordination between the partners who aim to achieve a common goal [1].

The mechanisms for planned coordination during complex tasks are still unclear [2,3]. One existing debate concerns the need to share high-level representations and intentions to engage in a joint action [4–8]. A growing number of behavioral data suggest that it may be necessary to share motor representations of both partners’ actions to efficiently couple and coordinate the joint task [9–13]. Recently, Vesper et al. [13] studied how two persons, who could not see each other, coordinate their actions during simultaneous jumps. The participants were first informed about the distances that their partner and they themselves had to jump, and they were asked to land as simultaneously as possible. It turned out that the participants who performed “easier” (i.e. shorter) jumps adapted their trajectories and timings to the lengths of their partners’ jumps. This result suggests that the participants used motor simulation of the partner’s actions to achieve the requested coordination. Simulating others’ actions while planning own actions would allow accurate coordination during social interaction [14]. The simulation would, however, be context-dependent [15], and in competitive conditions some participants might be able to prevent the simulation to improve their own performance [16].

The first indications of brain-level motor simulation of the partner’s action during real joint actions were obtained in an functional magnetic resonance imaging (fMRI) study where fronto-parietal areas were activated more strongly during complementary than imitative actions [17,18]. However, these results were challenged by a study showing stronger, rather than weaker, activation of the fronto-parietal areas during imitative actions [19], suggesting that the previous results reflected a stronger cognitive effort in the imitative condition. Kourtis et al. [20] investigated the brain areas involved in joint action in participants who lifted and clinked drinking glasses while their electroencephalogram (EEG) was measured; the task was performed either alone—unimanually or bimanually—or jointly, unimanually—with a partner. Event-related potentials associated with attention allocation (Anterior Directing Attention Negativity (ADAN) and Late Directing Attention Positivity (LDAP)) as well as motor activity (Motor Related Potential (MRP) and Contingent Negative Variation (CNV)) were influenced by the joint action: MRPs and CNVs were more similar in the joint action and in the bimanual condition than in the unimanual condition, supporting the hypothesis of a motor simulation of the partner’s action.

Similarly, Ménoret et al. [21] investigated how complementary actions modulate motor brain activity in two interacting participants. In their task, an actor was always grasping an object and displacing it in front of an observer, while the observers were passively observing (“observation” condition) or performing a complementary action later on (“interaction” condition). In both actor’s and observer’s brain, EEG beta oscillations time-locked to the onset of motor actions were suppressed more in the interaction than the observation condition. These modulations of motor-cortex activity were suggested to reflect representation of the others’ action during joint action. The results are in line with the finding that perturbing the activity of the primary motor cortex (M1) by transcranial magnetic stimulation (TMS) interferes tempo adaptation during live piano play (i.e. the melody part), but only when the complementary part (i.e. the bass-line part) had been rehearsed (i.e. existed in the pianist motor repertoire) [22].

During joint action, the partner’s actions thus seem to be represented in addition to own actions. However, previous studies have not addressed the specificity of such a representation with respect to joint action. Indeed, joint actions have been previously compared with solitary and “meaningless” actions [20,21], making it impossible to tell apart the role of the action’s goal and the role of the observation of the partner’s action, regardless of its relevance to the joint action.

Our goal was to investigate more precisely how human motor-cortex activity is modulated during joint actions. However, it is challenging to study brain activity during complex joint actions because the actions are typically continuous and because both participants act simultaneously so that motor activity related to own actions easily masks the activity related to simulation of the partner’s action. To obtain a robust and easily quantifiable brain signal, we combined a joint action task with median-nerve stimulation that was used to probe the functional state of the primary motor cortex as has been done previously [23]. Median-nerve stimuli applied to the wrist at intensities above the motor threshold elicit brief thumb twitches, but they also modulate the rolandic MEG ~20-Hz oscillations, first inducing suppression and then an increase (“rebound”) that originates mainly from M1 [24]. The rebound is thought to reflect an inhibitory or stabilized state of the motor cortex, promoting the maintenance of the *status quo* at the level of the effector [25–27], keeping the existing state and thereby preventing movement. The beta rebound can thus be used as an indicator of the functional state of the motor cortex, and it is already well-established that it is abolished during movement execution and suppressed during action observation [23] and motor imagery [28].

We used action observation associated with motor imagery (AO+MI) to make the task as natural and engaging as possible in the absence of overt motor activity (see [29] on the relevance of studying AO+MI): the participants were asked to imagine playing a ball maze game while watching a real play on video. This game can be played by one or two players by pulling strings to move a ball in a maze. Motor imagery is known to share common mechanisms with action execution [30], and Vesper et al. [31] have used a motor imagery task, adapted from their joint-jumps study [13], to confirm that the same simulation mechanisms are recruited during execution and imagination of joint actions. We varied both the goal of the actions (the ball-to-be-moved present or absent in the maze game) and the number of participants (1 or 2) to understand how those parameters influence motor simulation during joint action.

We expected to observe activation of the motor cortex during AO+MI and stronger motor-cortex involvement during motor imagery of goal-directed than non-goal-directed actions, as suggested by a previous action-observation study [32]. Additionally, we hypothesized that observation of a second participant’s action would induce stronger motor-cortex activity than the observation of an individual action. Such an effect could be specific to the joint action or it could be automatically triggered by the observation of a second hand. Finally, we expected the hemispheric dominance of the motor-cortex reactivity to give information about the effector-specificity of the motor simulation [20].

## Material & Methods

### 1 –Participants

Seventeen healthy participants took part in this experiment. One participant was discarded from the analysis because of absence of rolandic rhythms (see Analysis section). The presented results are based on 16 participants (mean age 25.1 yrs, range 19–35; 8 men, 8 women).

None of the participants reported any history of motor or neurological disorders. All were right-handed (scores from 0.65 to 1 at the Edinburgh handedness inventory [33]) and had normal or corrected-to-normal vision. The study was approved by the ethics committee of the Helsinki and Uusimaa Hospital District and all participants gave their written informed consent before the experiment. Participants were compensated monetarily for lost working hours and travel expenses. Due to national legislation regulating the ethics committees, we are not able to distribute the original data, but the anonymized subject-wise averaged MEG data are available by request (menoret@neuro.hut.fi, mathieu.bourguignon@aalto.fi).

### 2 –Stimuli and Tasks

The whole experiment lasted for about 1.5 hours, including the preparation, MEG measurement (40 min), and answering to a debriefing questionnaire.

**Fig 1:**
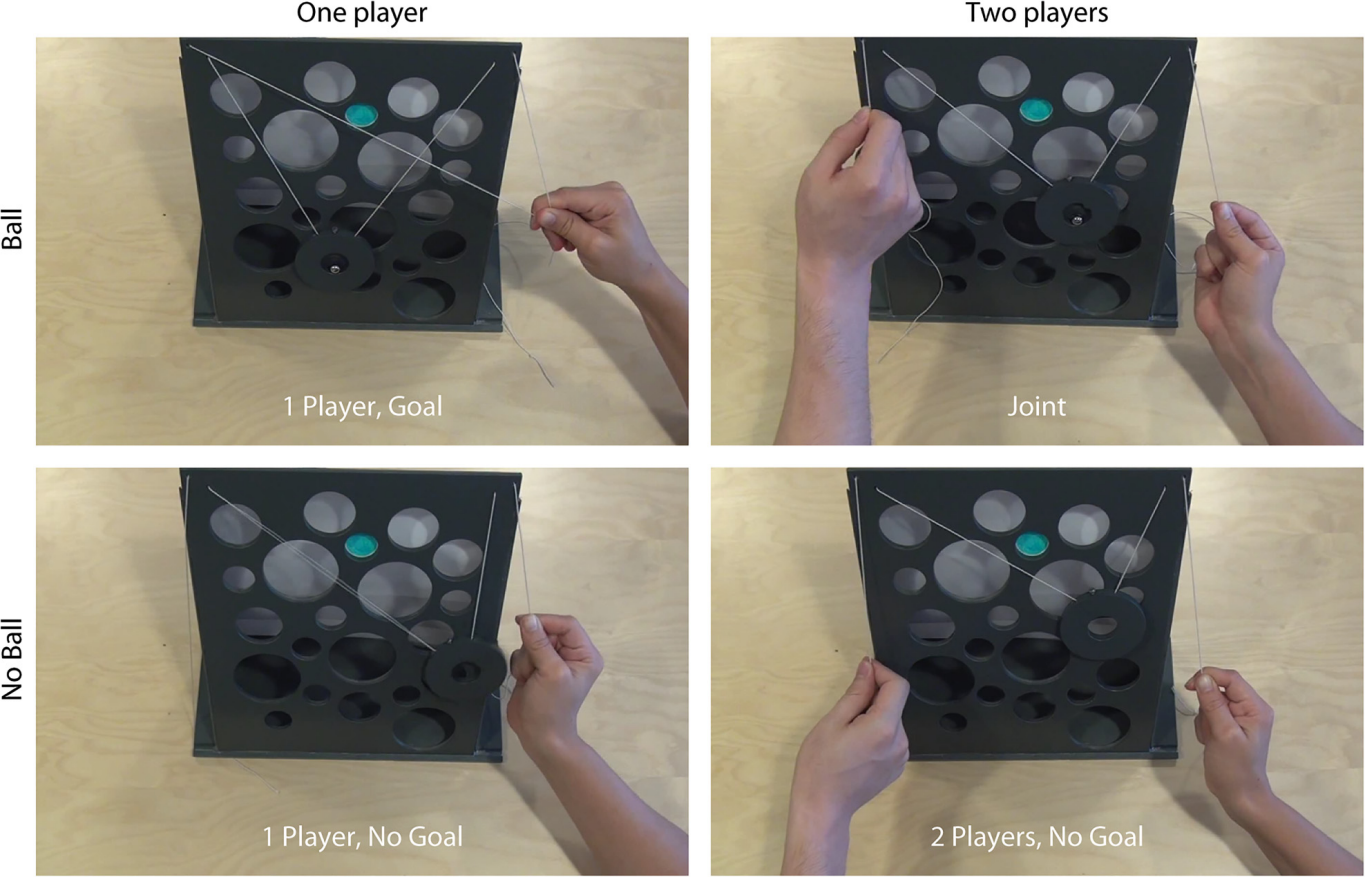
Experimental conditions. Selected frames extracted from the video clips representing the four motor-imagery conditions.

The participants’ task was to watch videos of a ball-maze game in a first person view (see Fig 1 and Supporting Information) while imagining “being the player” on the right. The goal of this game is to move a ball up on a wall full of holes, trying to avoid the holes to reach the destination (blue hole in Fig 1). The ball was placed inside a flat ring (outer diameter 70 mm, inner diameter 25 mm) suspended from two strings hanging from the corners of the game. By slowly pulling the strings, the ring (and the ball inside) could be moved through the maze. We selected this game because it can be played alone or with another player and because it requires a continuous coordination in pulling the strings to move the ring left or right.

The players used only their right hand to play.

Four game conditions were tested by varying the goal of the actions (i.e. ball present or absent) and the number of players (one or two players) in a 2 × 2 design: 2 Players–Goal (*Joint*), 2 Players–No Goal, 1 Player–Goal, 1 Player–No Goal. In the 2 Players, No Goal condition, the partner did not control the ring’s trajectory.

To obtain some experience of the task before the experiment, the participants played the game, alone and with the experimenter. The practice session included all 4 conditions (moving the ring without the ball and with the ball, both individually and with the partner) until the participants were able to finish the game twice per condition, which typically took about 5 min.

During the experiment, the participants were presented with videos depicting the 4 conditions, and they were asked to imagine performing with their right hand the movements of the player on the right. Each video lasted for 2 min 40 s, and blocks of similar duration with rest and own right-hand actions were also recorded. During the rest block, the participants relaxed and watched a static picture of the game (without hand), and during the action block, they manipulated a small eraser with the right hand at a regular pace.

The experiment comprised altogether 12 blocks (total duration 32 min = 12 × 2 min 40 s), so that each block was presented twice but in a counterbalanced order in all participants (Action, Rest, Video 1, Video 2, Video 3, Video 4, Video 4, Video 3, Video 2, Video 1, Rest, Action). Moreover, the order of the 4 videos was randomized across participants.

At the end of the experiment, the participants filled debriefing questionnaires, rating the difficulty of the motor-imagery task on a scale from 1 (easy) to 4 (impossible)) and their attention level on a scale from 1 (extremely attentive) to 5 (not attentive at all).

### 3 –Recordings

Brain activity was recorded with a 306-channel whole-scalp neuromagnetometer (Elekta Neuromag Oy, Helsinki, Finland) in a magnetically shielded room (Imedco AG, Hägendorf, Switzerland) in the MEG Core of the Aalto NeuroImaging, Aalto University. The signals were bandpass-filtered to 0.03–330 Hz and sampled at 1 kHz.

Four head-position-indicator coils were attached to the subject's scalp, and head coordinates were registered with a 3D digitizer by identifying the locations of the coils together with the locations of three anatomical landmarks (nasion and left and right preauricular points) and some additional points on the scalp. Vertical electro-oculograms (EOGs) were recorded during the experiment. The position of the subject's head inside the MEG helmet was continuously monitored via signals delivered by the head-indicator coils far beyond the frequency of interest for the brain signals.

During the experiment, the participants were seated comfortably in the MEG room, and their left and right median nerves were alternatingly stimulated by 0.2 ms constant-current pulses applied to the wrists once every 1.5 s, with intensities exceeding the motor threshold (median across individuals 8 mA, range 5–13 mA).

### 4 –Analysis

For each video, we quantified the mean displacement rate as follows. We first manually extracted, at every *δt* = 200 ms, the coordinates of (1) the small screw located on the top of the ring, and (2) the contact point between the string and the right thumb. The resulting time series of x- and y-coordinates were then low-pass filtered at 1 Hz, and the displacement rate *v* was obtained as $v=\left\langle \sqrt{{\left(x(t)-x(t-\delta t)\right)}^{2}+{\left(y(t)-y(t-\delta t)\right)}^{2}}\right\rangle /\delta t$.

As each 2 min 40 s long video was a concatenation of discontinuous trials, the time points corresponding to transitions between trials were not used in the estimation of the displacement rate.

The MEG data were first preprocessed using signal-space separation (SSS) method to suppress external interferences and to correct for head movements [34,35]; the SSS algorithm was implemented in the MaxFilter software (version 2.2; Elekta Neuromag Oy, Helsinki, Finland).

MEG data were analysed using Matlab 7.0 (MathWorks, Natick, MA) and FieldTrip [36], a free Matlab toolbox. The signals were notch-filtered at 50 Hz and harmonics, and they were segmented from –1000 ms before to 2000 ms after the median-nerve stimuli, separately for left- and right-hand stimulation. For each condition, the data from the two blocks were pooled before the analysis (52 left-hand and 52 right-hand stimuli for each block).

Segments with artifacts were detected and removed using the Fieldtrip visual artifact rejection method that displays, for each channel and trial, the variance and z-score values. We manually selected the outliers by visually inspecting the data. A discrete Morlet-wavelet decomposition was applied to each artifact-free trial (at least 90 segments per condition) by convolving the signal with complex Morlet wavelets characterized by a ratio between their mean and standard deviation in the Fourier domain of 7, a value that roughly corresponds to the number of cycles of the wavelet. Wavelet’s mean frequency was varied from 5 to 40 Hz in 1 Hz steps. Time–frequency power in this frequency range was then obtained as the square modulus of corresponding wavelet coefficients. A single time–frequency power map was then obtained for each gradiometer pair as the sum of the power of both gradiometers.

**Fig 2:**
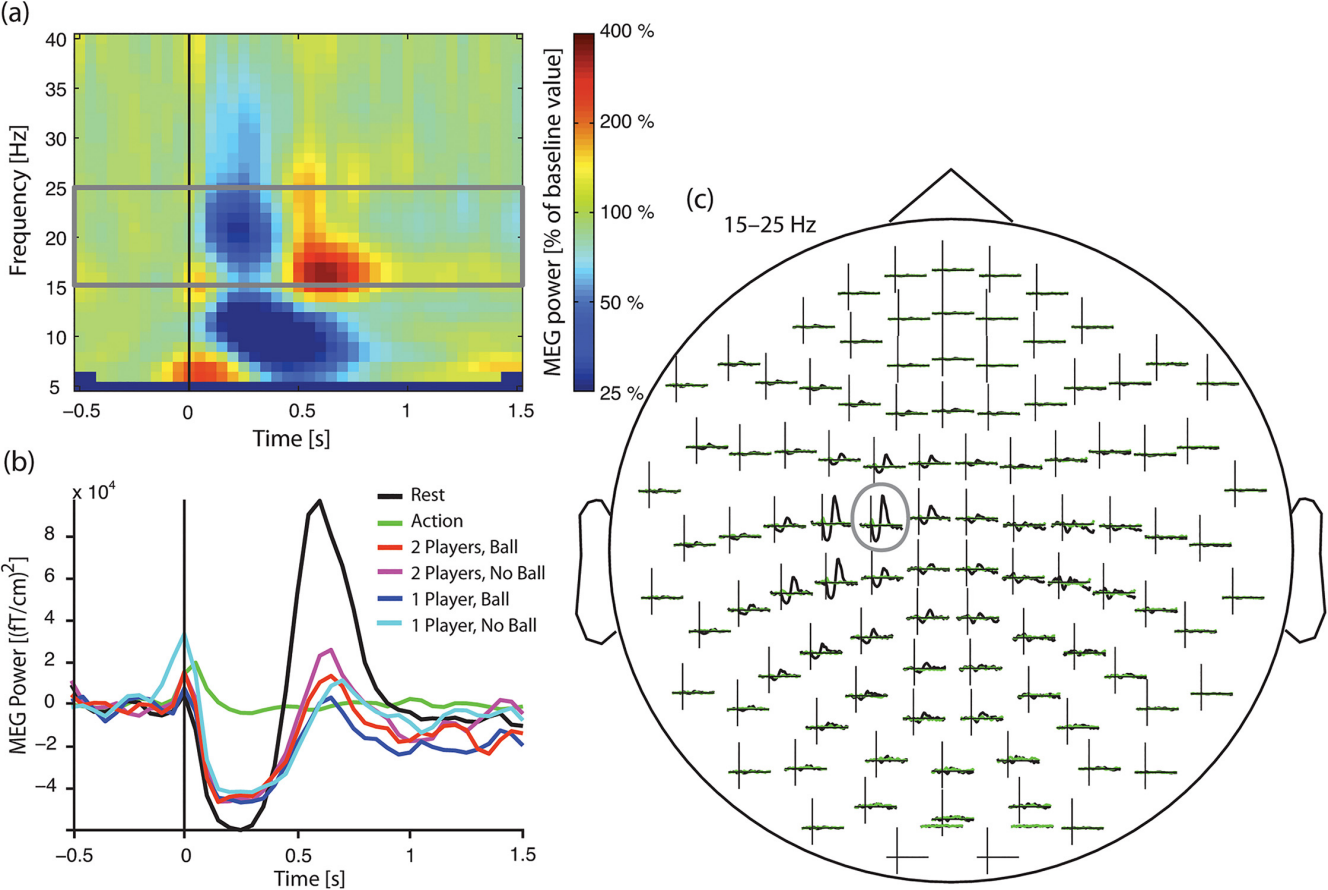
MEG power changes induced by median-nerve stimulation. Example taken from one subject who shows the evolution of the beta activity following the median-nerve stimulation (15–25-Hz frequency band in this subject) (a) Time–frequency representation in rest condition, time-locked to the onset of the median-nerve stimulation, for the left rolandic sensor showing the highest power modulation. For visualization purposes, the power is expressed in percentage of the baseline period chosen from –400 ms to –100 ms. (b) Power evolution for all the conditions in the most reactive ~20-Hz frequency band, at the most reactive sensor. (c) Spatial distribution of the power evolution in the most reactive ~20-Hz frequency band for the rest condition (black) and the action condition (green).

Fig 2 illustrates the reactivity of the beta oscillations in one subject, with a clear suppression of beta in the 15–25 Hz frequency range after the stimuli and with a rebound peaking about 700 ms after the stimulus.

The reactivity showed considerable inter-individual variability, some subjects displaying strong suppression but no rebound or, on the opposite, no suppression but strong rebound. Therefore, to better capture the group-level dynamics of the mu rhythm in the rest condition, time–frequency maps were averaged across a pre-selection of 9 sensors covering the SM1 cortex contralateral to the stimulation side. Individual maps were then normalized by their baseline value computed from –400 to –100 ms and averaged across subjects.

**Fig 3:**
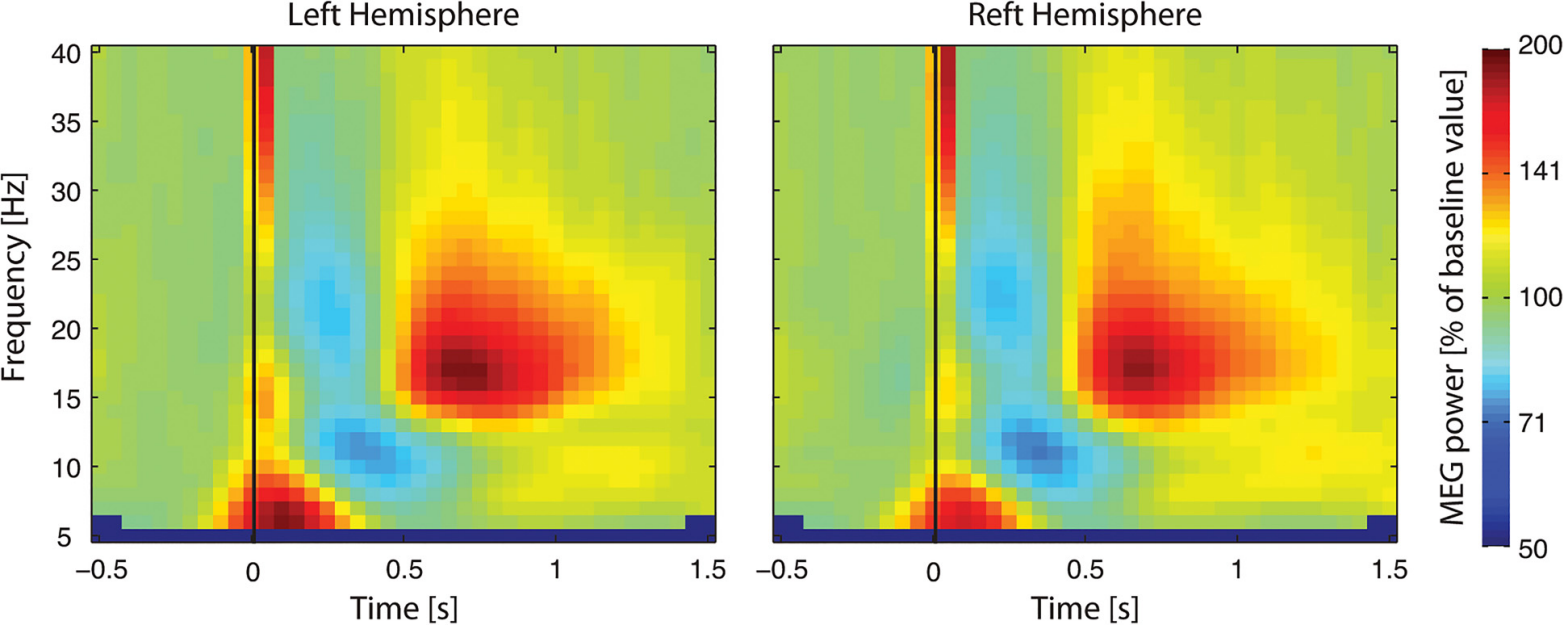
Group-level MEG power changes induced by median-nerve stimulation. Resting-state time–frequency maps averaged across a pre-selection of 9 sensors covering the SM1 cortex contralateral to the stimulation side, normalized by their baseline value evaluated from –400 to –100 ms, and averaged across subjects. The strong wide-band power enhancement peaking right after the stimulation reflects somatosensory evoked responses to median-nerve stimulation.

Fig 3 presents the ensuing group-level maps (during rest), time-locked to left and right median-nerve stimulation. The mu rhythm is first blocked, with a maximum suppression ~250 ms after the stimulus, and then enhanced, with a maximum at ~700 ms. On the basis of these maps, we defined the timing of the suppression as t_suppression_ = [100 400] ms, and the timing of the rebound as t_rebound_ = [400 900] ms.

On the basis of individual time–frequency power maps in the rest condition, we then visually identified, for each individual, the most-reactive 20-Hz frequency band in the rolandic region (*F*). When *F* could not be clearly identified, the default value of 15–30 Hz suggested by the group-maps was used. The ensuing *F* was 15–30 Hz (n = 8), 15–25 Hz (n = 5), 14–21 Hz (n = 1), 15–23 Hz (n = 1), 15–27 Hz (n = 1), and 17–30 Hz (n = 1).

Time–frequency power in each sensor was then averaged across this frequency band: *P*(*t*) = ⟨*P*(*f*,*t*)⟩|_*f*∈*F*_. We next calculated the power modulation (PM), i.e. the difference between suppression and rebound as $PM=\underset{t\in T_{\text{rebound}}}{\max}P(t)-\underset{t\in T_{\text{suppression}}}{\min}P(t)$. The contralateral rolandic sensor of maximum *PM* in the rest condition was then identified and used in further analyses. Subjects with too low mu rhythm modulation at this sensor, as reflected by relative PM defined by $\frac{\underset{t\in T_{\text{rebound}}}{\max}P(t)-\underset{t\in T_{\text{supression}}}{\min}P(t)}{\underset{t\in T_{\text{rebound}}}{\max}P(t)+\underset{t\in T_{\text{supression}}}{\min}P(t)}\times 100\%$ below 25% in both hemispheres were excluded from further analyses. One subject was excluded based on this criterion. For every condition and included subject, we then computed the motor-involvement index (MII) that we defined as

$$MII_{\text{task}}=\frac{PM_{\text{rest}}-PM_{\text{task}}}{PM_{\text{rest}}}\times 100\%.$$

MII is close to 0% if the rolandic rhythm is affected by the median-nerve stimulation as much as in the rest condition, suggesting the absence of motor involvement in the task, and close to 100% if the rolandic rhythm is unaltered following median-nerve stimulation, suggesting a strong motor involvement.

Source locations of the 20-Hz oscillations were identified in 3 subjects to confirm the previously well-established motor-cortex origin of the 20-Hz rhythm. Equivalent current dipoles of the unaveraged 15–30 Hz (the most-reactive 20-Hz frequency band for these 3 subjects) oscillations were identified based on signals of ~30 sensors covering the left rolandic area, using a spherical head model determined from subject’s own magnetic resonance images [37]. Sources were accepted if the field patterns appeared dipolar and if the dipoles explained at least 90% of the field variance. Fifty dipoles from separate oscillation cycles were superimposed on the individual MRI images.

### 5 –Statistics

Friedman two-way analysis of variance by ranks was performed on the behavioral ratings (attention and difficulty scores).

To test for a possible difference in the number of averaged trials between conditions, we performed a two-way ANOVA (Condition × Hemisphere) with the number of averaged trials as dependent variable.

To compare the MII values between the Action condition and the AO+MI conditions (mean across the four AO+MI conditions), a two-way ANOVA (Condition × Hemisphere) was performed with the MII values as dependent variable.

To compare the MII values measured in the four different AO+MI conditions, a three-way repeated-measures ANOVA (Hemisphere × Number of Players × Goal) was performed with the MII values as dependent variable. The Newman-Keuls test was used for post hoc comparisons. A significance level of p < 0.05 was chosen.

## Results

### Behavioral ratings

Friedman test revealed a statistically significant difference between conditions on the attention ratings (χ2 (3) = 26.4, p = 0.001) and on the difficulty levels (χ2 (3) = 7.9, p = 0.042). Post-hoc analyzes based on Wilcoxon test revealed that participants reported being statistically significantly more attentive to the videos when the ball was present within the ring than when it was absent (2.1 *vs*. 2.9; p = 0.003) and that motor imagery was easier when the ball was present than when it was absent (1.7 *vs*. 2.1; p = 0.036).

### Kinematics of the visual stimuli

Table 1 presents the displacement rates for both the ring and the experimenter’s right thumb in all four conditions. The displacement rates were on average 35% higher in 1 Player than 2 Players conditions. Moreover, the displacement rate was for the thumb (but not for the ring) about 35% higher in the Goal than the No Goal conditions.

**Table 1:** Mean ± SD displacement rates [pixels/s] of the ring and of the experimenter’s right thumb.

	1 Player		2 Players	
	Goal	No Goal	Goal	No Goal
ring	13.3 ± 7.5	14.6 ± 8.6	9.3 ± 5.6	10.5 ± 6.2
right thumb	21.1 ± 13.1	14.8 ± 11.4	15.2 ± 15.4	11.8 ± 7.2

### MEG results

The two-way ANOVA (Condition × Hemisphere) performed on the number of accepted trials revealed no effect of Condition (F(5,75) = 2.13; p = 0.07) or hemisphere (F(1,75) = 0.07; p = 0.79), and no interaction F(5,75) = 0.50; p = 0.78). Across subjects, conditions and hemispheres, the number of accepted trials was 100 ± 3 (mean ± SD).

**Fig 4:**
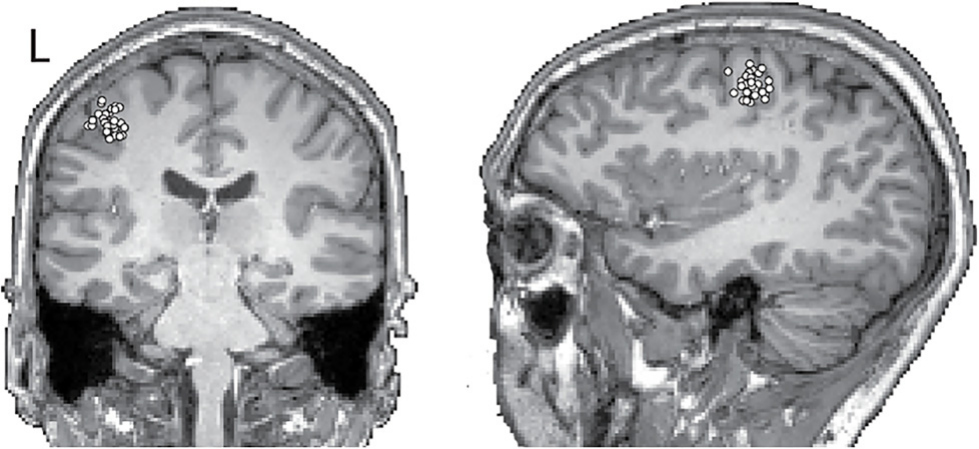
Left hemisphere sources for the 20-Hz activity in one subject. The dots represent 50 equivalent current dipoles, computed for different cycles of 20-Hz oscillations, superimposed on the subject’s magnetic resonance images. The dipoles cluster around the hand knob, which corresponds to the anatomically defined hand motor area.

Fig 4 displays the source locations of ~20-Hz oscillations for one subject. The sources are located in the precentral primary motor cortex, in the region of the hand knob, in agreement with previous studies [24,32,38].

**Fig 5:**
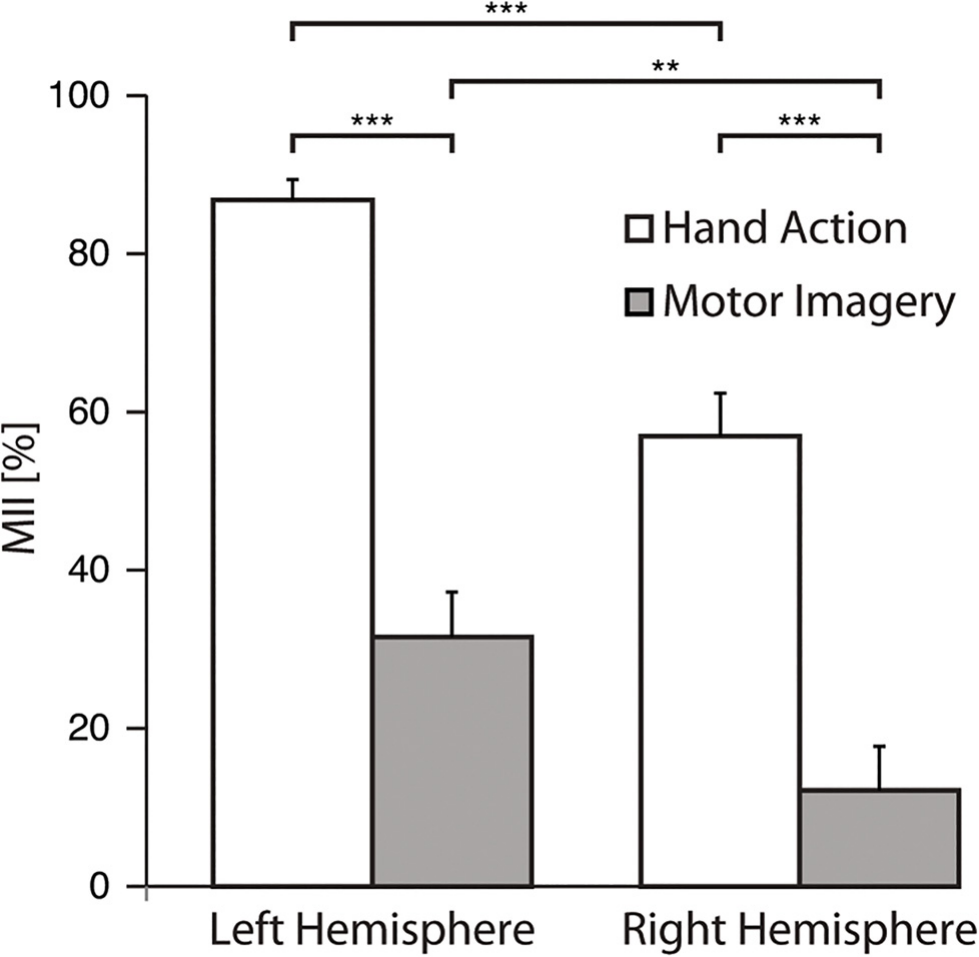
Motor involvement during action execution and AO+MI. Mean values of Motor Involvement Index (MII) for the AO+MI (averaged across all conditions) and hand action conditions over left and right hemisphere. Error bars represent standard error of the mean (SEM). **p < 0.01 and ***p < 0.001.

Fig 5 displays the MII values for the own-right-hand-action task and for the AO+MI conditions (mean across the four conditions) in both hemispheres. Two-way ANOVA (Condition × Hemisphere) revealed a main effect of Condition (F(1,15) = 154; p = 0.001) and of Hemisphere (F(1,15) = 37; p = 0.001). As it could be expected, the MII was stronger for own right-hand actions than for AO+MI, and it was stronger in the left than the right hemisphere (Fig 5).

**Fig 6:**
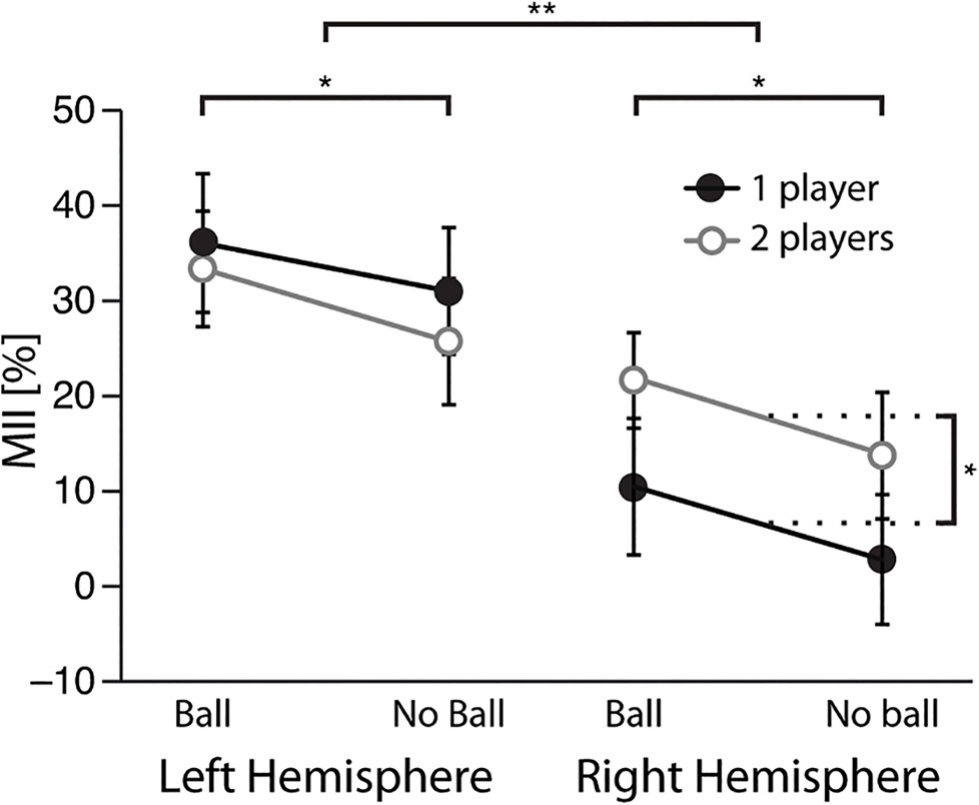
Motor involvement during AO+MI. Mean values of Motor Involvement Index for the four AO+MI conditions over left and right hemisphere. Error bars represent standard error of the mean (SEM). *p < 0.05 and **p < 0.01.

Fig 6 displays the MII values for all AO+MI conditions in both hemispheres. The three-way repeated-measures ANOVA (Hemisphere × Number of Players × Goal) revealed a main effect of Hemisphere (F(1,15) = 12.2, *p* = 0.003), a main effect of Goal (F(1,15) = 7.7, *p* = 0.014) and a statistically significant effect of the Hemisphere × Number of Players interaction (F(1,15) = 5.8; *p* = 0.029). Motor involvement was stronger in the left than in the right hemisphere during AO+MI (31% vs. 12%). Additionally, motor involvement was stronger during AO+MI for goal-directed (with ball) than non-goal-directed (with no ball) actions in both hemispheres (25% vs. 18%). The presence of the hand of the second player (i.e. partner) induced a significant stronger motor involvement in the right hemisphere (18% vs. 7%, p = 0.024) but not in the left hemisphere (30% vs. 34%, ns).

## Discussion

The present study aimed to advance our understanding of motor-cortex involvement during joint action. Action observation associated with instructed motor imagery was associated with activation of the observer’s primary motor cortex (that was the main source of the 20-Hz rolandic rhythm we were monitoring), mainly in the hemisphere contralateral to the viewed and imagined hand movements. This motor-cortex involvement was modulated by the goal of the actions but also, in the ipsilateral hemisphere, by the presence of the partner’s hand in the display, leading to a stronger motor involvement during motor imagery of joint action.

These results are in line with previous findings [15,20,21] on stronger motor-cortex activation during joint compared with individual actions. However, the present study sheds some new light on this effect, implying that it reflects an additive effect of two factors: the goal of the action and the observation of the partner’s actions, even when they are irrelevant to the task. This kind of distinction was not possible in previous studies that compared individual “meaningless” actions with joint “meaningful” actions. The enhanced involvement of the motor cortex is suggestive (1) of differential motor activity during goal-directed vs. non-goal directed actions, and (2) of shared motor representation of the partner’s actions, in addition to the participant’s own actions, during the joint action.

Enhanced activation of the motor cortex during observation of goal-directed actions has already been reported in a number of previous studies on both movement observation [39–41] (but see [42] for opposite results) and motor imagery [43]. Such results are in line with the existence of motor mirroring systems in the human brain, activated both during action execution and observation [for a review, see [44]]. In humans, such mirroring is thought to encode both goal-directed movements and motor acts and to facilitate understanding of others’ actions [44]. In addition to effector-specific motor representations, observation of goal-directed actions could activate also semantic representations of the actions [43,45,46].

Besides motor mirroring, motor recruitment might be modulated by the action complexity [47], attention allocation [48], and effort [49]. Our participants were, by self-report, more attentive and vigilant during the goal-directed conditions, feeling that the game with a ball was more engaging and required more precision (i.e. to follow the trajectory of the ball); they considered motor imagery more difficult in the non-goal directed conditions.

Finally, it is not likely that the stronger motor recruitment in Goal conditions would be related to differences in the kinematics between the visual stimuli. Indeed, although both the MII and the experimenter-hand displacement rate were higher in Goal than No Goal conditions, these two parameters showed an opposite behavior in One Player vs. Two Players comparison (smaller MII in the left hemisphere despite higher displacement rates of the ring and of the experimenter’s hand).

Interestingly, the observation of the partner’s hand (i.e. the second hand that was not imagined) modulated the motor-cortex activity, even when the partner’s actions were not relevant for the task, but this effect only occurred in the right hemisphere. It should be emphasized that the participants were specifically instructed not to imagine the partner’s action, and that in the “2 players, No Goal” condition, the partner’s movements had no impact on the ring’s trajectory. This result shows that observing the partner’s action activated the viewer’s motor system, distinct from activation associated with motor imagery. Moreover, a second hand in the display seems to have automatically activated the motor representation area of that hand as well. These results are not surprising considering the previous observations on motor mirroring in the human brain [23,50–52].

However, the right-hemisphere dominance of the effect was unexpected because the partner was using his right hand only to pull the string, which should have induced a stronger modulation over the contralateral hemisphere if only the location of the movement input would have been relevant. However, previous studies have demonstrated that healthy humans naturally prefer mirror-image imitation to imitation according to anatomical correspondence [53–55]. Accordingly, the human mirror-neuron system has been suggested to be preferentially recruited in a mirror-image manner [56]. Therefore, the stronger right-hemisphere involvement could either reflect a general right-hemisphere dominance of motor mirroring [57] or it could pertain to the location of the partner’s hand (that was present in the subject’s left visual field).

The motor activation in the ipsilateral hemisphere suggests that in our unimanual task the partner’s action could be represented in the same manner as bimanual actions, as if the participants themselves were playing with both hands. In their behavioral experiment, Vesper et al. [13] showed that, when participants jumped on one foot jointly with a partner or when they jumped individually with their both feet, the movement onsets were similarly influenced by the context of the jumps (i.e. different jump lengths). These results, together with those of Kourtis et al. [20] who showed that the CNV was equally strong for bimanual than joint unimanual actions, are suggestive of a similar preparation mechanism for joint action and interlimb coordination. However, in the present AO+MI task, it is impossible to disentangle whether the motor-cortex activation is purely automatic, triggered by the observation of the partner’s hand, or whether this mechanism could help the coordination of the joint action.

The current results provide some precision about the properties underlying motor simulation. Simulating others’ actions in our own motor system might help to coordinate the actions using the same mechanisms that exist for interlimb coordination. However, the motor activation induced by the mere presence of a hand in our visual field seems detrimental: if every observed movement automatically elicits an activation of the motor system, in dense social environments such as crowds, our motor system would be overloaded by such stimulations. In our experiment, this “second-player” effect might have been enhanced by the video and game settings where both players’ hands (the one to imagine and the partner’s) were viewed from first person perspective and in the same peripersonal space. A filtering mechanism is probably involved to suppress unnecessary motor simulation, and it could rely on the social relevance [15,58] and the location of the observed actions. For example, Kilner et al. [58] showed that the orientation of observed movements (towards or away) modulated observation-related brain activity: movements towards the participants induced stronger suppression of the rolandic 10-Hz MEG oscillations than movements away from them. Additionally, Brozzoli and collaborators [59] proposed that the “hand-centered representation of space” observed in monkeys [60,61] is essential to help our interactions with the environment by providing a reference between hands and the surrounding environment [59,62]. Such a system might represent the mechanism that filters the relevant social actions that occur in our peripersonal space.

## Conclusion

We have shown that both the goal of an observed action and the presence of a partner’s hand in the peripersonal space modulate motor-cortex activity, which is indicative of a representation of the partner’s action, in addition to the observer’s own, during joint actions. We suggest that motor imagery associated with movement observation represents a valuable method to study complex joint actions but also different social interactions, because it provides a good compromise between passive observation of social stimuli and real social interactions.

## Supporting Information

S1 Movie Video stimuli 1 Player, Goal. Video clip showed to the participants during the condition MI+AO 1 Player, Goal.
(MP4)

S2 Movie Video stimuli 1 Player, No Goal. Video clip showed to the participants during the condition MI+AO 1 Player, No Goal.
(MP4)

S3 Movie Video stimuli 2 Players, Goal. Video clip showed to the participants during the condition MI+AO 2 Players, Goal (Joint action).
(MP4)

S4 Movie Video stimuli 2 Players, No Goal. Video clip showed to the participants during the condition MI+AO 2 Players, No Goal.
(MP4)
